# 71. Increasing Trends in Multimorbidity and Polypharmacy Over a 5-Year Period in People Living with HIV in the United States

**DOI:** 10.1093/ofid/ofab466.071

**Published:** 2021-12-04

**Authors:** Misti Paudel, Girish Prajapati, Erin K Buysman, Swarnali Goswami, Jianbin Mao, Kimberly McNiff, Princy N Kumar

**Affiliations:** 1 Optum, Eden Prairie, MN; 2 Merck & Co., Inc., Rahway, NJ; 3 Merck & Co., Inc., Kenilworth, NJ (Author was working under an internship in partnership with The University of Mississippi, University, MS), University, Mississippi; 4 Acceleron Pharma, Cambridge, Massachusetts; 5 Georgetown University Hospital, Potomoc, MD

## Abstract

**Background:**

Advances in antiretroviral therapies (ART) have resulted in people living with HIV (PLWH) living longer with higher risk for age-related comorbid conditions and polypharmacy. The aim of this study was to describe trends in comorbidity and comedication burden in PLWH over a 5-year time period.

**Methods:**

A retrospective analysis of commercial and Medicare Advantage enrollees from the Optum Research Database was conducted. Annual cohorts of PLWH were constructed for each calendar year from 2014-2018 and included adults (≥ 18 years) with ≥ 1 pharmacy claim for an ART or medical claim with an HIV/AIDS diagnosis code (index date=earliest claim date in each calendar year). Continuous health plan enrollment of 12 months prior to (baseline), and 30 days after index date was required for each annual cohort. Comorbidities were identified using ICD-9/10 diagnosis codes from medical claims during baseline period and comedications from pharmacy/medical claims in the 90-days prior to index using National Drug Codes. Charlson Comorbidity Index (CCI) was computed excluding HIV/AIDS. P-for-trend values accounting for clustering by patients across calendar years were assessed.

**Results:**

Overall, 14,222 - 20,249 PLWH who were enrolled in commercial (80.7%-65.4%) or Medicare Advantage (19.3%-34.6%) plans were identified in 2014 - 2018 calendar years. Notable trends in demographics of PLWH were observed across years, including increases in mean age (48.9 to 52.4 years), proportion of females (17.2% to 20.3%) and Black race (25.9% to 29.0%), all p-trend< 0.001. Mean CCI scores increased across years (0.72 to 0.93), p-trend< 0.001. Multimorbidity (≥2 non-HIV conditions) and polypharmacy (≥ 5 non-ART medications) prevalence increased over 5 years (Figure 1). Hypertension, hyperlipidemia, neuropsychiatric conditions and Type 2 diabetes mellitus were the most prevalent comorbid conditions with statistically significant upward trends in prevalence across years (Figure 1).

**Conclusion:**

Multimorbidity and polypharmacy are common in PLWH and have been increasing in prevalence over the past 5 years. Study findings highlight the importance of an individualized approach to care for a diverse PLWH population, in order to minimize drug-drug interactions and adverse events and thereby improve patient outcomes.

Figure 1. Comorbidity and Comedication Trends by Index Year among People Living with HIV

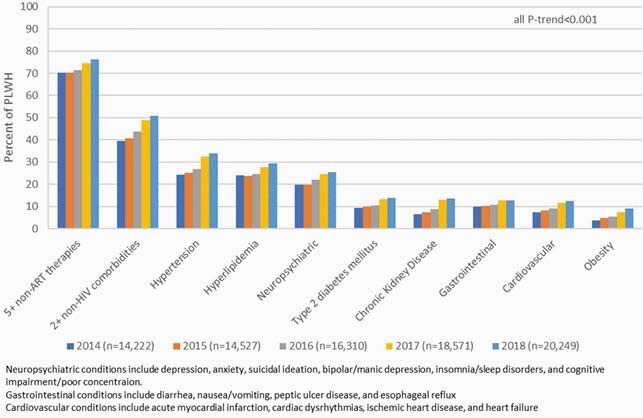

**Disclosures:**

**Misti Paudel, PhD**, **Merck** (Other Financial or Material Support, This study was funded by Merck & Co.) **Girish Prajapati, M.B.B.S., MPH** , **Merck & Co., Inc.** (Employee, Shareholder) **Erin K. Buysman, MS**, **Merck & Co., Inc.** (Other Financial or Material Support, I am an employee of Optum, which was contracted by Merck to complete the research described in this abstract) **Jianbin Mao, PhD**, **Merck & Co.** (Employee, Shareholder) **Kimberly McNiff, MPH**, **Merck** (Other Financial or Material Support, Merck funded the research project) **Princy N. Kumar, MD**, **Amgen** (Consultant)**Eli Lilly** (Grant/Research Support)**Gilead** (Consultant, Grant/Research Support, Shareholder)**GSK** (Consultant, Grant/Research Support, Shareholder)**Merck** (Consultant, Grant/Research Support, Shareholder, Honoraria)

